# Unusual Oligomeric Laccase-like Oxidases from Ascomycete *Curvularia geniculata* VKM F-3561 Polymerizing Phenylpropanoids and Phenolic Compounds under Neutral Environmental Conditions

**DOI:** 10.3390/microorganisms11112698

**Published:** 2023-11-03

**Authors:** Zhanna V. Renfeld, Alexey M. Chernykh, Boris P. Baskunov, Anastasya S. Gaidina, Nina M. Myasoedova, Anna D. Egorova, Olga V. Moiseeva, Sophya Yu Gorina, Marina P. Kolomytseva

**Affiliations:** G.K. Skryabin Institute of Biochemistry and Physiology of Microorganisms, Federal Research Center “Pushchino Scientific Center for Biological Research of the Russian Academy of Sciences”, Prosp. Nauki 5, 142290 Pushchino, Russia; zhanna.renfeld@yandex.ru (Z.V.R.); achernykh@rambler.ru (A.M.C.); baskunov@ibpm.pushchino.ru (B.P.B.); anneteshebanova@mail.ru (A.D.E.); omoiseeva@rambler.ru (O.V.M.); sofya.gorina.1991@mail.ru (S.Y.G.)

**Keywords:** alkaliphilic laccase, laccase-like oxidase, transformation, polymerization, phenylpropanoids, phenolic compounds, proton channel, ascomycete, neutral environmental conditions

## Abstract

The unique oligomeric alkaliphilic laccase-like oxidases of the ascomycete *C. geniculata* VKM F-3561 (with molecular masses about 1035 and 870 kDa) were purified and characterized for the first time. The ability of the enzymes to oxidize phenylpropanoids and phenolic compounds under neutral environmental conditions with the formation of previously unknown di-, tri-, and tetrameric products of transformation was shown. The possibility to obtain industrially valuable compounds (dihydroxybenzyl alcohol and hydroxytyrosol) from caffeic acid using laccase-like oxidases of *C. geniculata* VKM F-3561 has been shown. Complete nucleotide sequence of the laccase gene, which is expressed at the peak of alkaliphilic laccase activity of the fungus, and its promoter region were determined. Based on the phylogenetic analysis of the nucleotide sequence, the nearest relationship of the isolated laccase gene with similar genes of fungi of the genera *Alternaria*, *Bipolaris*, and *Cochliobolus* was shown. Homologous model of the laccase structure was predicted and a proton channel was found, which was presumably responsible for the accumulation and transport of protons to T2/T3-copper center in the alkaliphilic laccase molecule and providing the functional activity of the enzyme in the neutral alkaline environment conditions.

## 1. Introduction

In recent years, the interest in a search and study of atypical fungal laccases active in a neutral or slightly alkaline medium is constantly growing, mostly because the new technologies such as cell platforms for the biosynthesis of pharmacologically and industrially valuable compounds, biosensors, biofuel cells for implantable devices, 3D nanodevices for biomedical purposes, synthesis of C-N heteropolymer dyes, and etc., usually use media with pH close to neutral [1,2,3,4,5,6,7]. Having a high redox potential and, as a result, a broad substrate specificity, fungal laccases have significant advantages over bacterial laccases and laccase-like enzymes. However, the most typical fungal laccases are functionally active at acidic pH, which makes their use much more difficult in technological processes occurring under neutral and slightly alkaline environmental conditions.

Only about ten of more than three hundred laccases studied to date are known to be most active against phenolic substrates at slightly alkaline medium (pHopt ≥ 7.0): laccases from basidiomycetes *Coprinus cinereus* [8,9], *Moniliophthora perniciosa* FA553 [10], *Rhizoctonia praticola* 93a [11], *Rhizoctonia solani* [12], *Pleurotus pulmonarius* CCB19 [13], *Panaeolus papilionaceus* CBS 630.95, and *Coprinus friesii* CBS 629.95 [14], as well as from ascomycetes *Scytalidium thermophilum* ST26 [15], *Acremonium murorum* [16], and *Myrothecium verrucaria* 24G-4 [17]. 

In addition, some laccases exhibit their maximum activity at pH close to neutral (pH 6.0–7.0). These are laccases of the basidiomycetes *Coprinopsis cinerea* [18], *Flammulina velutipes* [19], and *Rigidoporus lignosus* [20], as well as the ascomycete laccases from *Myceliophthora thermophila* CBS 117.65 [21], *Melanocarpus albomyces* VTT D-96490 [22], *Aspergillus niger* [23,24], and *Sordaria macrospora* k-hell [25]. 

Recently, our team has found fungi producing oxidases that are the most active in relation to phenolic compounds and phenylpropanoids under slightly alkaline environmental conditions (at pH 7.0–9.0): *Rhizoctonia solani* VKM F-895, *Myrothecium verrucaria* VKM F-3851, *Myrothecium roridum* VKM F-3565, and *C. geniculata* VKM F-3561 [26,27]. Alkaliphilic laccase of the fungus *M. roridum* VKM F-3565 was isolated and characterized [28]. A novel proton channel has been observed in its molecule as well as in molecules of known alkaliphilic fungal laccases, which provide the transfer of protons to the T2/T3-copper cluster necessary for functional activity of the enzyme under neutral and alkaline environments [28].

In this work, we study the unique highly oligomeric alkaliphilic laccase-like oxidases from the fungus *C. geniculata* VKM F-3561, which are capable of polymerizing phenolic compounds and phenylpropanoids under neutral conditions with formation of novel di-, tri-, and tetrameric products having potential for further application in pharmacology and industry.

## 2. Materials and Methods

### 2.1. Chemicals

All reagents used in the work were of an analytical grade. Syringaldazine (4-hydroxy-3,5-dimethoxybenzaldehyde azine) and phosphoric acid were from Sigma-Aldrich (St. Louis, MO, USA); 2,2′-azino-*bis*(3-ethylbenzothiazoline 6-sulfonic acid) (ABTS) and 2,6-dimethoxyphenol were from Fluka (Buchs, Switzerland); ferulic acid, coniferyl alcohol, Coomassie brilliant blue, and glycerol were from Serva (Heidelberg, Germany); peptone and soy flour were from Difco (Tucker, GA, USA). The DE-52 carrier was from Whatman (Florham Park, NJ, USA). Other media and columns were from GE Healthcare Life Sciences (Marlborough, MA, USA). Molecular mass markers (ovalbumin, bovine serum albumin, alcohol dehydrogenase, apoferritin, thyreoglobulin, and dextran blue) used in gel filtration were from Sigma-Aldrich (St. Louis, MO, USA). All other reagents were purchased locally. 

### 2.2. Cultivation Procedure 

The fungus *Curvularia geniculata* VKM F-3561 was obtained from the All-Russian collection of microorganisms (VKM) of IBPM RAS. The fungal strain was maintained on slant glucose-potato agar (g/L: glucose—10; fresh finely grated potatoes—200; agar—20) at 29 °C, stored at 4 °C. 

The inoculum was grown in 750 mL flasks with stirring (200 rpm) for 6 days at 29 °C in 100 mL of soy-glycerol medium [26,28,29]. The medium contained (g/L): NH_4_NO_3_—0.2, KH_2_PO_4_—0.2, K_2_HPO_4_—0.02, MgSO·7H_2_O—0.1, peptone—0.5, soy flour—0.5, and glycerol—4.0. The obtained homogenized mycelium was added to 750 mL flasks at the rate of 10 mL of the mycelium per 100 mL of the mineral medium containing the mixture of ground seeds of 5 cereal crops (barley, buckwheat, oats, wheat, and rye) in the ratio 1:1 (*w*/*w*) as a sole source of carbon and energy in various concentrations (20–120 g/L). If necessary, the inducers of laccase activity (CuSO_4_, 2,6-dimethoxyphenol, 2,6-dimethylphenol, ferulic, *p*-coumaric, caffeic, or tannic acids) were also added in various concentrations (0.25–3.00 mM) on the second day of cultivation. Mycelium was cultivated with active stirring at 200 rpm, 29 °C. 

### 2.3. Laccase Activity Assay

Laccase activity in culture liquid was determined spectrophotometrically at 436 nm by the rate of ABTS oxidation, taking into account the molar extinction coefficient *ε*_436_ = 29,300 M^−1^ cm^−1^ [30] using a UV-160 spectrophotometer (Shimadzu, Kyoto, Japan) in a quartz cuvette with an optical path length 10 mm at 25 °C in 20 mM Na-acetate buffer (pH 5.0) containing 1 mM ABTS. The average rate of conversion of 1 μmol of substrate per minute by 1 mg of the enzyme or 1 mL of the culture liquid was taken as a unit of laccase activity.

### 2.4. Purification of Laccase-like Oxidases of C. geniculata VKM F-3561

The laccase-like oxidases were purified from a 20-day culture liquid filtrate of *C. geniculata* VKM F-3561 grown during submerged cultivation in the above-mentioned mineral medium in the presence of 100 g/L of the mixture of ground seeds of 5 cereal crops. The fungus culture liquid was preliminarily centrifuged at 15,344× *g* for 10 min at 4 °C to remove the mycelium. The obtained supernatant was subjected to chromatography, consisting of 4 successive stages: on DE-52 carrier (180 mL) with a linear gradient of 0–0.5 M NaCl in 1800 mL of 20 mM Na-acetate buffer pH 5.0 (buffer A), on Q-Sepharose carrier (40 mL) with a linear gradient of 0–0.33 M NaCl in 300 mL of buffer A, on Resource Q (6 mL) with broken gradient of 0–2%, 2–4%, 4–10%, 10–20%, 20–100% of 1 M NaCl in 60 mL of buffer A, on Superdex 200 (120 mL) with 0.1 M NaCl in buffer A. Final oxidase solution was desalted and concentrated applying ultrafiltration with Millipore membrane (10 kDa) and stored at 4 °C for further application. 

### 2.5. Enzyme Characterization

The native mass of the oxidases was determined by gel filtration using a Superdex 200 column (120 mL) equilibrated with 20 mM Na-acetate buffer (pH 5.0) containing 0.1 M NaCl. The elution was carried out with the same buffer at a rate of 0.9 mL/min. The volume of fractions was 0.5 mL. Proteins of known molecular mass (ovalbumin—45 kDa, bovine serum albumin—66 kDa, alcohol dehydrogenase—150 kDa, apoferritin—443 kDa, thyreoglobulin—669 kDa) and dextran blue (2000 kDa) were used as standards.

The subunit mass of the oxidases and their deglycosylated forms was determined using SDS electrophoresis in 7% polyacrylamide gel according to the modified method of Laemmli [31]. Thermo Scientific™ PageRuler™ protein molecular weight markers (10–180 kDa) were used as standards. The gel was stained with Coomassie brilliant blue G-250 [32].

The deglycosylation of the purified native oxidases was performed using Endoglycosidase F1 and Endoglycosidase F3 of the Native Protein Deglycosylation Kit (NDEGLY-1KT, Sigma-Aldrich, St. Louis, MO, USA). The procedure was carried out during 24 h according to the manufacturer’s instructions. Deglycosilation of the enzymes was then confirmed by 7% SDS-PAGE.

Protein concentration was determined by the Bradford method using bovine serum albumin (BSA) as a standard [33]. 

The pH optimum of the purified oxidase activity was determined spectrophotometrically in the universal Britton–Robinson buffer [15,34] in the range of pH 2.0–9.0 at 25 °C as described previously [35]. A total of 0.1 mM ABTS, 2,6-dimethoxyphenol, ferulic acid, and coniferyl alcohol or 0.01 mM syringaldazine were used as substrates. The reaction was initiated by addition of an enzyme preparation into a cuvette, and the absorption of the substrate or the forming product was recorded at the appropriate wavelength [35].

The T-optimum of the oxidase activity was determined in the temperature range from 15 to 80 °C in the reaction with 0.1 mM ABTS in 20 mM Na-acetate buffer (pH 5.0) [30].

UV-visible absorption spectra of the purified oxidases of *C. geniculata* VKM F-3561 were determined in 20 mM Na-acetate buffer (pH 5.0) or 50 mM Tris-HCl buffer (pH 7.2) in a quartz cuvette with an optical path length 10 mm at 25 °C in the range of 180–800 nm using a Shimadzu UV-2501PC spectrophotometer (Kyoto, Japan).

Apparent *K*_m_ and *V*_max_ as well as *k*_cat_ values were calculated as described earlier using the molar extinction coefficients of the substrates or the corresponding formed products [35]. To determine the kinetic parameters of the oxidases, 20 mM Na-acetate buffer (pH 5.0) was used in the reaction with ABTS and 50 mM Tris-HCl buffer (pH 7.2) with the other substrates, taking into account the molar extinction coefficients of the corresponding products [35]. Calculation of each kinetic parameter was performed using at least three experiments. All kinetic constants were obtained with error < 10%.

### 2.6. Isolation and Identification of Phenylpropanoid and Phenolic Compound Intermediates Produced by the Oxidases of C. geniculata VKM F-3561

The 20-day cultural liquid of the fungus *C. geniculata* VKM F-3561 obtained at the peak of alkaliphilic laccase activity during submerged cultivation of mycelium in a mineral medium with 100 g/L of a mixture of ground seeds of 5 cereal crops and prepared as describe above, was used for an oxidase preparation.

The transformation of various phenolic compounds and phenylpropanoids was carried out in a reaction mixture containing 2 mM of an aromatic substrate (caffeic acid, ferulic acid, 4-hydroxyphenylacetic acid, *p*-coumaric acid, coniferyl alcohol, eugenol, guaiacol, 3-methoxycatechol, 2,6-dimethoxyphenol, vanillic acid, isovanillin, or 2-(4-hydroxyphenyl ethanol)), the enzyme preparation (750 U/mL in reaction with ABTS [30]) in 50 mM Tris-HCl buffer (pH 7.2). The reaction was started by enzyme addition and the reaction mixture was incubated for 4 h at 29 °C. The reaction was stopped by addition of ethyl acetate to the reaction mixture. The products of the phenylpropanoid transformation were extracted from the reaction mixture with ethyl acetate [36], were isolated and analyzed by Thin layer chromatography (TLC, 60 F_254_ Silicagel plates (Merck, Darmstadt, Germany)) and MS-spectrometry (using LCQ Advantage MAX low-resolution quadrupole mass spectrometer (Thermo Finnigan, San Jose, CA, USA)), as described earlier [35,36]. The intermediates of the phenylpropanoid and phenolic compound transformation were identified using standards, as well as using data from the mass spectrum library and published studies.

### 2.7. Identification of Nucleotide Sequence of the Laccase Gene Expressed at the Peak of Alkaliphilic Laccase Activity of the Fungus C. geniculata VKM F-3561

Isolation of genomic DNA of the fungus *C. geniculata* VKM F-3561 was carried out as described previously [28]. 

To isolate total RNA, the fungus *C. geniculata* VKM F-3561 was cultivated under optimal conditions for twenty days until the peak of alkaliphilic laccase activity was detected. The obtained fungal mycelium was separated from the culture liquid by centrifugation at 5000× *g* for 10 min at 4 °C and was dried. A total of 100 mg of the mycelium was dispersed in liquid nitrogen, 1 mL of the reagent ExtractRNA (Evrogen, Moscow, Russia) for extracting total RNA was added, and the further isolation of total RNA was performed according to manufacturer’s recommendations. A total of 500–800 ng of the obtained total RNA was taken for the synthesis of a complementary DNA strand using a Mint cDNA synthesis kit (Evrogen, Moscow, Russia) according to the manufacturer’s protocol. 

PCR amplification of the central fragment of the laccase gene was performed using the universal degenerate primers Lac2FOR and Lac3REV (Supplementary Materials Table S1) [37], as described previously [28]. Amplification was performed using a MiniCycler PTC-150 PCR amplifier (MJ Research, Deltona, FL, USA). 

Flanking regions of the laccase gene were obtained by SON-PCR [38]. The reaction mixture for SON-PCR (25 µL) contained 2 µL of the corresponding GSP1 primer (10 µM) (Supplementary Materials Table S1), 5 µL of Screen Mix solution (Evrogen, Moscow, Russia). A total of 10 ng of the gDNA was used as a template DNA at the first stage. At the second stage, 2 μL of the product of the first reaction diluted 50 times and 2 μL of the corresponding GSP2 primer (10 μM) were used as a template (Supplementary Materials Table S1). The modified SON-PCR program was applied as described previously [28].

PCR amplification of the full laccase gene from the gDNA of *C. geniculata* VKM F-3561 was performed using the gene-specific primers F3561-20d-Lcc-F and F3561-20d-Lcc-R (Supplementary Materials Table S1). The reaction mixture (25 μL) contained 1 μL of each of the primers (10 μM), 2 μL of the gDNA, 2.5 μL of 10× Tersus Plus buffer (Evrogen, Moscow, Russia), 0.5 μL of 50× dNTP mixture (NEB, UK), 0.5 μL of 50× Tersus polymerase (Evrogen, Moscow, Russia). The PCR program included pre-denaturation step (3 min, 95 °C), 30 amplification cycles (45 s, 95 °C; 45 s, 59–60 °C depending on primer *T*_m_; 3 min, 72 °C) and final elongation step (5 min, 72 °C). 

The resulting DNA fragments were purified using the Cleanup Standard kit (Evrogen, Moscow, Russia), cloned into pAL2-T vector (Evrogen, Moscow, Russia) according to the manufacturer’s protocol, and sequenced using a service of the Evrogen company (Moscow, Russia).

### 2.8. Analysis of the Obtained Sequences

Nucleotide and amino acid sequences were analyzed using the NCBI Blastn and NCBI Blastp web services [39]. The search for start and stop codons and the analysis of the exon-intron structure were performed using the FGENESH web service (Softberry, Mt Kisco, NY, USA) [40]. The signal peptide was found using the SignalP-5.0 web service [41]. The NCBI CDD web service was used to search for conserved amino acid sequences [42]. Possible N-glycosylation sites were determined using the NetNGlyc 1.0 web service [43]. The search for motifs corresponding to known regulatory elements in the promoter region of the laccase gene was carried out using the Geneious Prime 2023.1.1 program (Biomatters Inc., Auckland, New Zealand). Known motifs were used to search for regulatory elements [44,45,46]: HSE (heat shock responsive element)—NGAANNTTCN and NGAAN; MRE (metal responsive element)—TGCRCNC; XRE (xenobiotic responsive element)—CACGCW and KCRYGS; ACE—HWHNNGCTGD and NTNNHGCTGN; ACE1 (activates laccase transcription in response to copper induction)—TACNGCTGA; nucleotide motifs TGGGTT and ATATC; ARE (antioxidant stress response element)—TGACNNNGC; STRE (stress response element)—CCCCT; creA (cAMP mediated glucose repression)—SYGGRG; NIT2 (Nitrogen repression response element)—GATA GATA TATCDH; pH-dependent element PacC—GCCARG; elements involved in transcription regulation—TATA (TATAWAW) and CAAT (GGNCAATCT); recognition site for Sp1 transcription factor—GGGCGG. 

### 2.9. Nucleotide Sequence of the C. geniculata VKM F-3561 Laccase Gene

The GenBank accession number for the laccase gene sequence reported in this paper is OR250480.1.

### 2.10. Homology Modelling and Structural Analysis

The search of the closest homologues of the studied laccase was performed using the BLAST web service [39] and the RCSB PDB database (on 4 April 2023; https://www.rcsb.org/). The crystal structures of the laccases of *Myceliophthora thermophila* (RCSB PDB: 6F5K), *Thielavia arenaria* (RCSB PDB: 3PPS), and *Melanocarpus albomyces* (RCSB PDB: 1GW0) were selected as templates for homology modeling of the structure of *C. geniculata* VKM F-3561 laccase by the Modeller 10.1 software package [47]. A hundred initial structures were calculated, and the best structure based on the lowest DOPE score was chosen. The models were validated using the verification server SAVES v6.0 (on 18 April 2023; https://saves.mbi.ucla.edu/). The root mean square deviation (RMSD) between atoms of the main chain of the model and templates was calculated to refine the model. Visualization and analysis of the obtained models was carried out in PyMol 2.5.0 [48].

The p*K*_a_ values of ionizable side chain groups were calculated using the PROPKA v3.4.0 software package [49]. The electrostatic potential was calculated using PDB2PQR 2.1. with the AMBER force field [50] and the APBS program [51]. The obtained data were visualized using the APBS plugin implemented in PyMol. 2.5.0 [48].

### 2.11. Phylogenetic Analysis 

For phylogenetic analysis, the DNA and mRNA sequences encoding known laccases as well as amino acid sequences of the laccases with known structure were obtained from GenBank and RCSB PDB databases. The alignment was performed with the BioEdit version 7.0.9.0 software [52] using the ClustalW algorithm (default parameters) with a number of bootstrap replications equal to 1000. The trees for nucleotide sequence alignment and for amino acid sequence alignment were constructed by the MEGA6 software [53] using the Maximum Likelihood and the Neighbor-Joining statistical methods, respectively. All positions containing gaps and missing data were eliminated.

## 3. Results and Discussion

### 3.1. Optimization of Cultivation Conditions for Alkaliphilic Laccase Production by the Fungus C. geniculata VKM F-3561

It has been shown that the seeds of cereals increase the alkaliphilic laccase activity of the fungus *C. geniculata* VKM F-3561 during submerged cultivation in a mineral medium [54]. 

**Figure 1:**
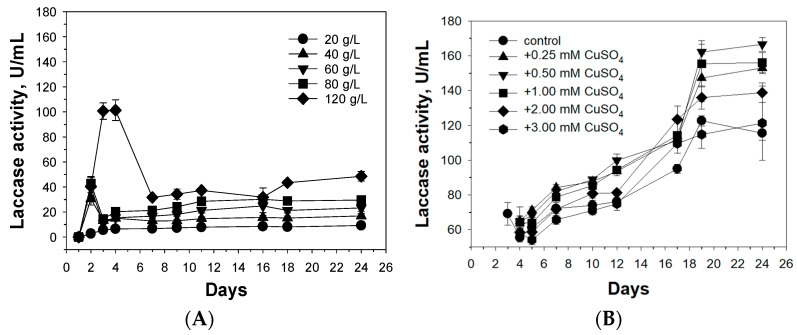
Dynamics of laccase activity of the fungus *C. geniculata* VKM F-3561 during submerged cultivation in the presence of various concentrations of the mixture of ground seeds of 5 cereals as sole sources of carbon and energy (**A**), as well as in the presence of 100 g/L mixture of the ground seeds and various concentrations of copper sulfate (**B**).

The study of laccase activity of the fungus during submerged cultivation in the presence of different concentration of the mixture of ground seeds of five cereal crops in the mineral medium led us to find the optimal conditions (120 g/L) for maximal laccase production (Figure 1A). In this case, the appearance of two peaks of laccase activity was observed: laccases produced by mycelium at the beginning of cultivation with activity at acidic pH region and laccases of a later peak (on the 18th–24th days of cultivation) containing only unusual laccases, the most active in a neutral and alkaline medium (Figure 1A). However, further work was carried out with 100 g/L of the ground seed mixture, since higher concentrations led to an increase of medium density that complicated protein purification.

The presence of the most active inducer of known fungal laccases, divalent copper ions [55], in the culture liquid of the fungus *C. geniculata* VKM F-3561 at concentrations up to 1 mM led to an increase of the fungal laccase activity by only 25–36% compared to the control (Figure 1B). Increasing the copper sulfate concentration to 3 mM inhibited the laccase activity of the fungus.

Aromatic compounds (2,6-dimethoxyphenol, 2,6-dimethylphenol, ferulic acid, *p*-coumaric acid, caffeic acid, tannic acid) did not induce the production of *C. geniculata* VKM F-3561 laccases or inhibit the laccase activity of the fungus during submerged cultivation.

### 3.2. Purification of the Alkaliphilic Laccase-like Oxidases of C. geniculata VKM F-3561

Laccase-like oxidases of the fungus *C. geniculata* VKM F-3561 were purified from the culture liquid obtained under optimal conditions after a 24-day submerged cultivation of the mycelium in a mineral medium with 100 g/L of a ground seed mixture of five cereals (Supplementary Materials Table S2). As a result of purification, two laccase-like oxidases were obtained: oxidase I (1.3 mg) and oxidase II (3.7 mg) with specific activities of about 15.4 U/mg and 22.9 U/mg and purification degrees of 51.3 and 76.3 times, respectively.

**Figure 2:**
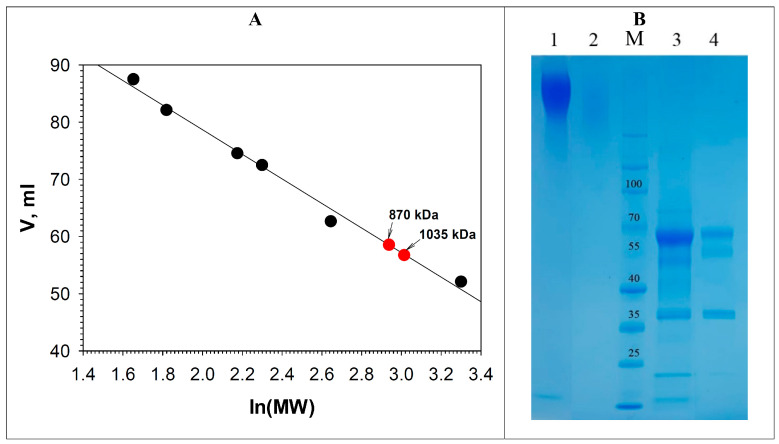
Determination of molecular ((**A**), by gel filtration on Superdex 200) and subunit ((**B**), by SDS-electrophoresis (7% PAGE)) masses of the purified fungal laccase-like oxidases of *C. geniculata* VKM F-3561. Here, 1035 kDa and 870 kDa are approximate molecular masses of the oxidase I and the oxidase II, respectively. The masses of the standards (45 kDa—ovalbumin, 66 kDa—bovine serum albumin, 150 kDa—alcohol dehydrogenase, 443 kDa—apoferritin, 669 kDa—thyreoglobulin, and 2000 kDa—dextran blue) are shown in black circles. 1—The glycosylated oxidase I, 2—the glycosylated oxidase II, M—standards, 3—the deglycosylated form of the oxidase I, 4—the deglycosylated form of the oxidase II.

According to the results of gel filtration of the purified oxidases of *C. geniculata* VKM F-3561 using a Superdex 200 column (120 mL), both enzymes had unusually high molecular masses (Figure 2): about 1035 kDa for oxidase I and about 870 kDa for oxidase II, which significantly exceeded the molecular masses of all known fungal and bacterial laccases [55,56]. 

SDS electrophoresis of *C. geniculata* VKM F-3561 oxidase samples boiled for 5 min in buffer containing SDS showed a putative dissociation of native oxidase molecules into fragments with molecular masses above 200 kDa (Figure 2B). 

Preliminary deglycosylation of the samples and subsequent 5 min boiling in the presence of SDS led to final dissociation of the initial molecules into subunits with a mass about 63 kDa for both enzymes (Figure 2B).

According to the obtained results, it can be assumed that the formation of oxidase oligomers of *C. geniculata* VKM F-3561 involves ionic bonds that hold approximately half of the molecules to each other, and glycosidic residues on the surface of subunits that promote the formation of primary smaller oligomeric complexes. Thus, both oxidases of *C. geniculata* VKM F-3561, in addition to unusual alkaliphilic properties, have an atypical oligomeric structure, which makes these objects interesting for further comprehensive study.

Laccases and multicopper oxidases with an oligomeric structure and high molecular mass are much less common in nature than the monomeric ones.

Thus, for example, a heterooligomeric laccase with a native molecular mass of about 390 kDa of the ascomycete *Podospora anserine* [57], an oligomeric laccase of the bacterium *S. anulatus* (SaSL) with a molecular mass of 235–300 kDa consisting of six or eight subunits [58], and a laccase-like enzyme from the green soil alga *Tetracystis aeria* with a molecular mass of about 240–250 kDa and six monomers [59,60], were reported.

It should be noted that there are no data concerning larger fungal and bacterial laccases than mentioned above. However, large enzymes were found among the other multicopper oxidases. For example, the phenoxazinon synthase of actinomycete *Streptomyces antiticus* had two forms of the enzyme, one with a high molecular mass of about 900 kDa and another one a low molecular mass of about 200 kDa with subunits about 100 kDa [61]. Later, X-ray diffraction analysis also showed the presence of two oligomeric forms of this enzyme: dimeric and hexameric [62]. Another multicopper oxidase MhMCO from the thermophilic bacterium *Marinithermus hydrothermalis* with laccase activity was crystallized as a dodecamer consisting of four trimers forming a closed spherical structure [63]. 

### 3.3. T-, pH Optima, and Stability of Isolated Oxidases

The study of the activity of the purified enzymes in the temperature range from 15 to 80 °C using ABTS as a substrate showed that both oxidases of *C. geniculata* VKM F-3561 had maximum activity at 70 °C, rather rare optimum temperature among fungal laccases [55,64].

The pH optimum of the ABTS oxidation by purified oxidases was in the strongly acidic pH range (2.0–2.8) (Table 1; Supplementary Materials Figures S1 and S2), as for the most known fungal laccases, which was explained by the nature of the substrate [55,65,66]. 

At the same time, the maximum activity of the studied oxidases with all tested phenolic compounds and phenylpropanoids was observed in the pH range of 6.8–7.5 depending on the substrate (Table 1; Supplementary Materials Figures S1 and S2), which was not typical for the most fungal laccases, showing maximum activity in an acidic environment. Activity at neutral or slightly alkaline pH is a demanded property for a number of modern biotechnological processes [7,55,67,68,69,70,71,72].

The isolated laccase-like oxidases of *C. geniculata* VKM F-3561 completely retained their activity after 7 days of incubation at pH 5.0 and pH 7.2, both at 4 °C and at room temperature. It is possible that such a high stability of the unusual blue copper oxidases of *C. geniculata* VKM F-3561 is associated with their oligomeric properties. 

Currently, there are two reports on the isolation of laccases from the fungi *Curvularia* sp. [73] and *Curvularia trifol* [74]. Both laccases were maximally active in an acidic medium, in contrast to the alkaliphilic laccase-like oxidases of the fungus *C. geniculata* VKM F-3561 isolated in this work. The laccase of *C. trifol* [74] had a molecular mass of about 92.3 kDa, which was also different from the oxidases of *C. geniculata* VKM F-3561 (1035 kDa and 870 kDa).

### 3.4. Spectral Properties of Isolated Laccase-like Oxidases 

**Figure 3:**
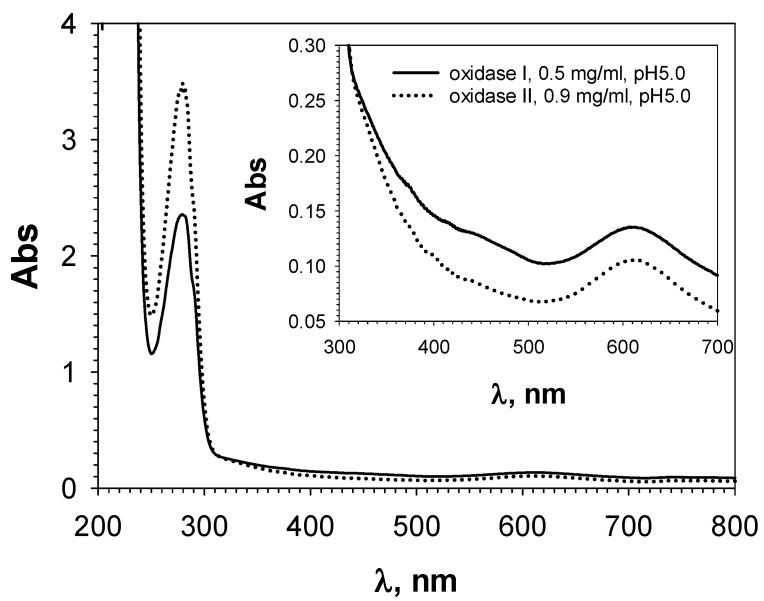
UV-vis absorption spectra of the purified glycosylated oxidases of *C. geniculata* VKM F-3561 in 20 mM Na-acetate buffer at pH 5.0.

The absence of an absorption peak of the T3 copper atom (330 nm) and the presence of additional arms in the region of about 372–374 nm, 450 nm, and a small peak at 409–414 nm were noted for both oxidases of *C. geniculata* VKM F-3561 (Figure 3, Table 2).

At the same time, the absorption peak of the T1-copper of the isolated oxidases of *C. geniculata* VKM F-3561 was in the region of 610 nm, which was typical for the most known laccases [35,55,75]. Similar spectral properties were observed for the alkaliphilic laccases of the ascomycetes *M. roridum* VKM F-3565 [28] and *Melanocarpus albomyces* [76], as well as for the alkaliphilic laccase of the basidiomycete *Rhizoctonia praticola* [11] and the plant alkaliphilic laccase from *Rhus vernicifera* [77,78], in which the T3 peak (330 nm) also disappeared or was smoothed, and new peaks appeared in the longer wavelength region of 360–450 nm (Table 2). It is assumed that the smoothing and shifting of the T3 absorption peak to longer wavelengths is due to the presence of a bound oxygen molecule in the trinuclear copper cluster [76,77].

**Table 2:** Spectral characteristics of known alkaliphilic laccases of fungi and plants.

Organisms	pH_opt_ with SG	T3-Peak, 330 nm	Modified T3-Peak, nm	T1- Peak, nm	References
*Coprinus cinereus*	7.0	no	no	614	[9]
*Rhizoctonia praticola*	7.4	smooth	370, 420	610	[11]
*Rhizoctonia solani* RS22	7.0	no	no	602	[12]
*Rhus vernicifera*	7.0, 8.5 *	no	370, 420	614	[77,78]
*Melanocarpus albomyces*	6.0–7.0	no	360, 450	600	[76]
*Myceliophthora thermophila* CBS 117.65	6.0–7.0	no	no	589	[21]
*Myrothecium roridum* VKM F-3565	7.8	no	370, 440	587	[28]
*Curvularia geniculata* VKM F-3561					
oxidase I	7.5	no	372, 411, 450	610	the present work
oxidase II	7.0	smooth	374, 409–414, 450	611	the present work

*—pH optima of oxidation of *p*-phenylenediamine and 2.6-dimethyphenol, respectively.

### 3.5. Kinetic Properties of the Isolated Laccase-like Oxidases

The isolated blue oxidases of the fungus *C. geniculata* VKM F-3561 were inactive with bilirubin and ascorbate, which are the substrates of bilirubin (EC 1.3.3.5) and ascorbate (EC 1.10.3.3) oxidases belonging to the same family of multicopper oxidases as laccases. However, the investigated enzymes showed the ability to oxidize ABTS (in an acidic environment) and phenolic substrates of laccases in an atypical neutral environment, which suggests that they are laccases (Table 1). 

The oxidase I of *C. geniculata* VKM F-3561 showed the highest specificity constant value for syringaldazine (*k*_cat_/*K*_m_ = 5375.7 min^−1^ µM^−1^), the elevated specificity was observed for ABTS (*k*_cat_/*K*_m_ = 38.4 min^−1^ µM^−1^) and 2,6-dimethoxyphenol (*k*_cat_/*K*_m_ = 33.0 min^−1^ µM^−1^), the reduced specificity for coniferyl alcohol (*k*_cat_/*K*_m_ = 13.8 min^−1^ µM^−1^), and the lowest specificity for ferulic acid (*k*_cat_/*K*_m_ = 1.3 min^−1^ μM^−1^) (Table 1). The oxidase II of *C. geniculata* VKM F-3561, as well as oxidase I, showed the elevated specificity for syringaldazine (*k*_cat_/*K*_m_ = 892.2 min^−1^ µM^−1^) and ABTS (*k*_cat_/*K*_m_ = 55.3 min^−1^ µM^−1^), the reduced specificity for ferulic acid (*k*_cat_/*K*_m_ = 17.4 min^−1^ μM^−1^) and coniferyl alcohol (*k*_cat_/*K*_m_ = 11.8 min^−1^ μM^−1^), the worst substrate for the oxidase II was 2,6-dimethoxyphenol (*k*_cat_/*K*_m_ = 7.8 min^−1^ μM^−1^). Nevertheless, isolated enzymes were able to oxidize phenylpropanoids (precursors of pharmaceutically valuable lignans) in a neutral medium.

### 3.6. Transformation of Phenylpropanoids and Phenolic Compounds by Laccase-like Oxidases of C. geniculata VKM F-3561

Laccases are able to oligomerize phenylpropanoids and phenolic compounds with the formation of lignans and their analogs, which are of pharmacological value due to their potential carcinolytic, cytostatic, antioxidant, antibacterial, fungicidal, antiparasitic, and other properties [7,28,35]. The use of laccases in the biosynthesis of pharmacologically valuable polymers based on cell platforms requires a high level of laccase activity in a neutral medium (cytosol).

Alkaliphilic laccase-like oxidases of the fungus *C. geniculata* VKM F-3561 oxidized caffeic and ferulic acids, guaiacol, 3-methoxycatechol, 2,6-dimethoxyphenol, and coniferyl alcohol in a neutral medium, but did not oxidize 4-hydroxyphenylacetic acid and eugenol (Table 3). 

The transformation of 2,6-dimethoxyphenol by the enzyme preparation almost completely proceeded to the formation of a dark-colored high-polymer compound insoluble in water. A similar insoluble product was also observed during the enzyme oxidation of guaiacol. 

Applying preparative TLC and mass spectrometry, 18 products of the tested compounds oxidation using laccase-like oxidases of the fungus *C. geniculata* VKM F-3561 were isolated and identified (Table 3). Ferulic acid was transformed into one trimer with [M+H]^+^637 and one tetramer with [M+H]^+^736. Two putative dimers with the same masses ([M+H]^+^341) and different mass spectra were found during coniferyl alcohol oxidation. Caffeic acid was transformed into dihydroxybenzyl alcohol (*m*/*z* = 140) and hydroxytyrosol (*m*/*z* = 154). Guaiacol oxidation resulted in the formation of five condensation products of two initial molecules with [M-H]^−^245, [M+H]^+^247, [M+H]^+^261, [M-H]^−^271, and [M-H]^−^275, as well as three trimers with [M+H]^+^369, [M-H]^−^455, and [M-H]^−^473 and one tetramer with [M+H]^+^489. 3-Methoxycatechol was transformed into two dimers (with [M+H]^+^317 and [M-H]^−^289) and one product of putative methylation of the parent compound with [M-H]^−^181. All oligomeric products were isolated and identified for the first time.

It is known that dihydroxybenzyl alcohols (*m*/*z* = 140) can be used in industry as precursors for the synthesis of composite materials, molecular sensors, and dendrimers, which are often used as encapsulating agents for the transfer of drug compounds for therapeutic and diagnostic purposes in medicine [79,80]. It has also been previously shown that hydroxytyrosol (*m*/*z* = 154) is one of the most powerful known antioxidants, exhibiting anti-inflammatory, antibacterial, and cardioprotective properties [81]. In the present work, the biosynthesis of such compounds by laccases from caffeic acid is shown for the first time.

### 3.7. Identification and Phylogenetic Analysis of the Nucleotide Sequence of the Laccase Gene of C. geniculata VKM F-3561 Expressed at the Peak of Alkaliphilic Laccase Activity

Degenerate primers Lac2FOR and Lac3REV complementary to highly conserved regions of the T2- and T3-copper centers of known laccases were used to amplify a 905 bp-fragment from the cDNA of the fungus *C. geniculata* VKM F-3561 isolated at the peak of alkaliphilic laccase activity. Blast analysis of the obtained nucleotide sequence showed 76% homology to the corresponding region of the laccase genes from fungi of the genera *Fusarium*, *Verticillium*, and *Colletotrichum*.

Based on the obtained partial nucleotide sequence of the laccase gene, pairs of the nested primers were designed (Supplementary Materials Table S1). They were used in two rounds of SON-PCR to obtain fragments of 1067 and 295 bp in size of the 5’-end as well as 635 and 1227 bp in size of the 3’-end. Analysis of the obtained sequences and subsequent amplification using genomic DNA as a template resulted in a fragment of 3189 bp containing 3 introns with a total length of 157 bp (Supplementary Materials Figure S3).

The putative translated protein sequence consisted of 600 amino acid residues and contained a 25 residue signal peptide with an ALS-YE cleavage site. The mature form of the protein included 575 amino acid residues. The molecular weight of the protein calculated using the Expasy web service (Compute pI/Mw, SIB Swiss Institute of Bioinformatics) was about 63 kDa, which corresponds to the mass of the dominant bands of the isolated deglycosylated laccases obtained during their electrophoresis. According to the analysis of the deduced amino acid sequence using the CDD database, the structure of the expected laccase contained three sequentially arranged cupredoxin-like domains, which are typical for the most fungal laccases. In addition, four highly conserved motifs (HXHG, HXH, HXXHXH, and HCHXXXHXXXXM/L/F) responsible for copper binding in the active site and typical for the copper-containing laccase family were identified in the resulting sequence (Supplementary Materials Figure S3). Seven putative N-glycosylation sites (N-X-S/T) were identified in protein sequence of the laccase of *C. geniculata* VKM F-3561.

**Figure 4:**
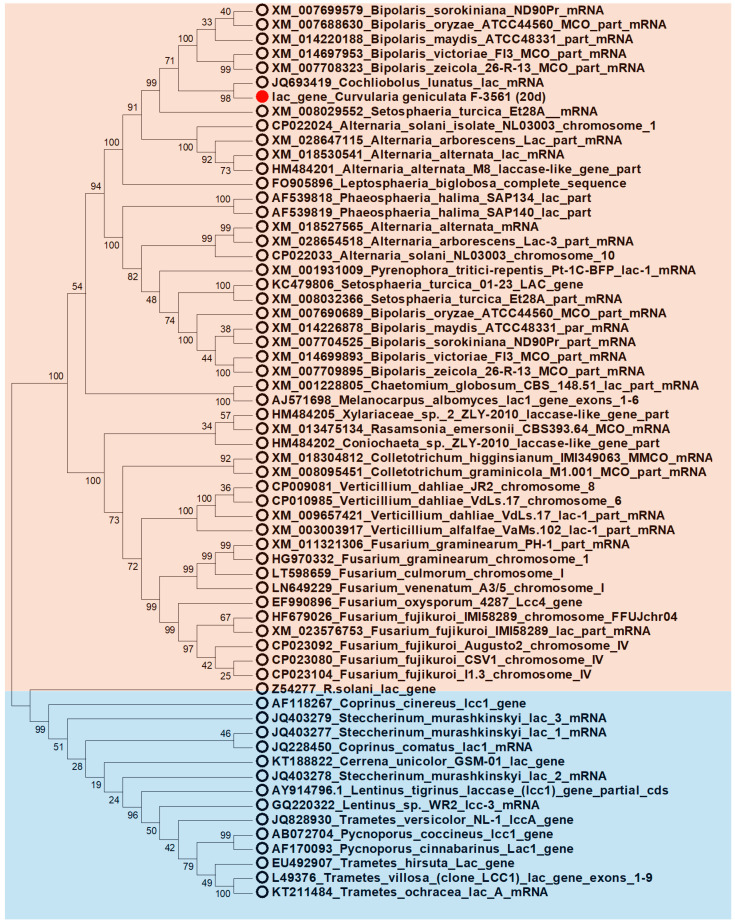
Phylogenetic relationships between the laccase gene of *C. geniculata* VKM F-3561 and the genes of known laccases inferred under the Maximum Likelihood criterion. Bootstrap values are placed near the corresponding nodes. Ascomycetes are marked in light red, basidiomycetes are in light blue. The sequence of the laccase gene of *C. geniculata* VKM F-3561 is marked with a red circle.

Phylogenetic analysis of the obtained nucleotide sequence of the laccase gene of *C. geniculata* VKM F-3561 showed the closest relationship with the nucleotide sequences of the genes that presumably encode laccases or laccase-like enzymes of fungi of the genera *Cochliobolus*, *Bipolaris*, and *Alternaria* (Figure 4). 

**Figure 5:**
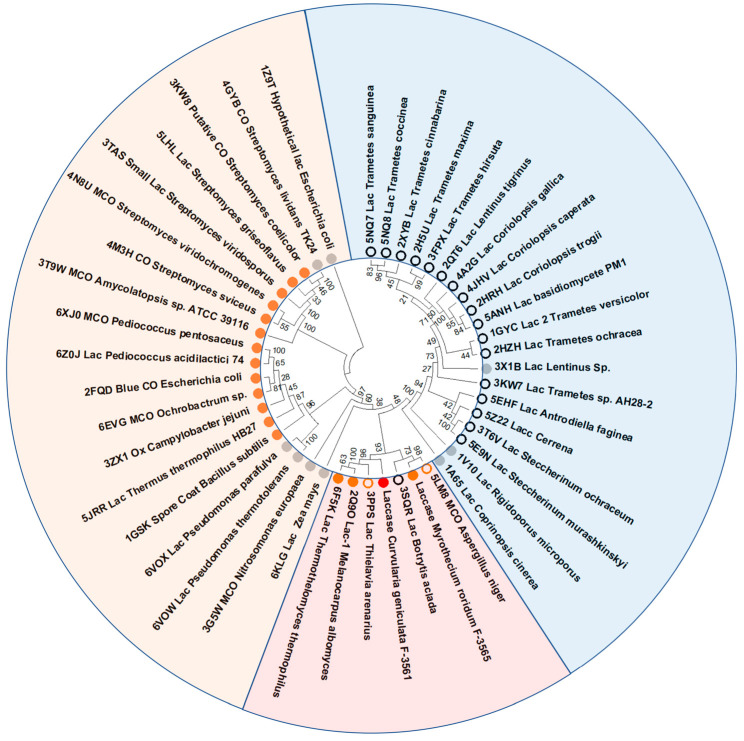
Phylogenetic relationships between the laccase from *C. geniculata* VKM F-3561 and all laccases with known structure based on the alignment of their amino acid sequences (RCSB PDB acc. numbers of proteins are indicated). The bootstrap values (consensus support, %) are displayed on the tree. The red filled circle marks the sequence of the laccase of *C. geniculata* VKM F-3561, the orange filled circles mark sequences of laccases which are more active with phenolic compounds at pH ≥ 7.0. The orange empty circles indicate the laccases, which are more active at pH values around 6.0. The laccases with maximum activity at acidic conditions (pH ≤ 5.0) are marked by the black empty circles, and the laccases with unknown pH optimum are marked by the grey filled circles. The laccases from ascomycetous fungi are marked in light red shadow, the basidiomycetous laccases are marked in light blue shadow, and the bacterial laccases are marked in light orange shadow.

Phylogenetic analysis of the deduced amino acid sequence of the laccase encoded by the isolated gene and the amino acid sequences of all laccases with a known structure showed the greatest relationship with laccases and laccase-like enzymes of ascomycetes, including the recently isolated and characterized alkaliphilic laccase of the ascomycete *M. roridum* VKM F-3565 [28] (Figure 5). Thus, ascomycete laccases occupy an intermediate position between basidiomycete and bacterial laccases, and, according to the literature data, they have an average redox potential of T1-copper [82,83].

### 3.8. Regulatory Elements in Promoter Region of the Laccase Gene of C. geniculata VKM F-3561

**Figure 6:**
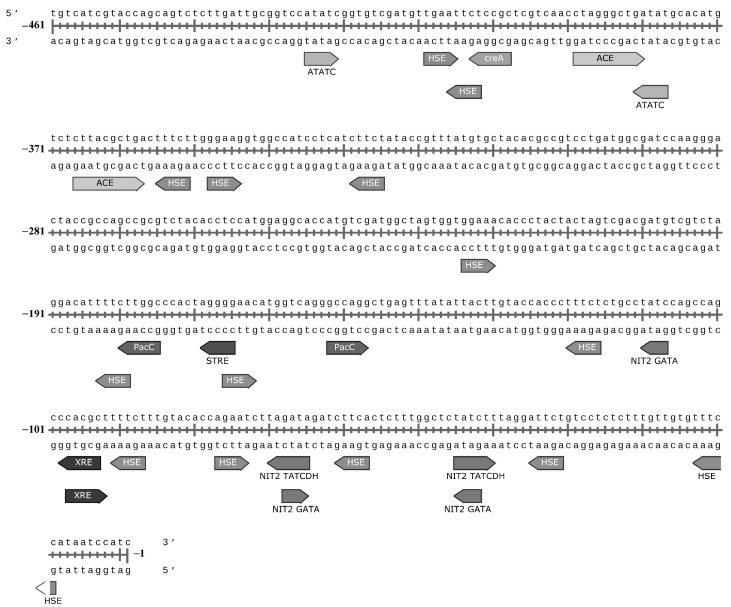
Regulatory elements in the promoter region of the laccase gene of the fungus *C. geniculata* VKM F-3561 isolated from mRNA at the peak of the fungal alkaliphilic laccase activity. HSE—heat shock responsive element, XRE—xenobiotic responsive element, ACE—element activating gene transcription in response to monovalent metals induction), STRE—stress response element, CreA—cAMP mediated glucose repression, NIT2—nitrogen repression response element, pH dependent element PacC, ATATC-element.

The N-terminal promoter region (−1–461 nucleotides) of the *C. geniculata* VKM F-3561 laccase gene expressed at the peak of the fungal alkaliphilic laccase activity contained 29 regulatory elements (Figure 6) found in the promoter regions of known laccase genes [44,45,46].

Among them, 14 HSE sites were observed: nine elements in the reverse direction 5′–3′ (−11–15, −33–37, −59–63, −89–93, −118–122, −181–185, −327–331, −353–357, and −404–408) and five elements in the forward 3′–5′ (−75–79, −164–168, −222–226, −346–350, −404–408).

The studied promoter region contained 3 NIT2 GATA sites (two in the 5′–3′-direction (−44–47, −109–112) and one site in the 3′–5′-direction (−72–67), while bidirectional NIT2 GATA sites were at a distance of 20 bp. Also in the promoter region, two bidirectional NIT2 TATCDH sites (−47–42 and −67–72) were found at a distance of 20 bp between each other. Moreover, two element pairs in areas −42–47 and −67–72 (one NIT2 GATA and one NIT2 TATCDH in each) overlapped at the same location.

The promoter region of the laccase gene of *C. geniculata* VKM F-3561 contained two PacC binding sites near the gene in two opposite directions (−54–49 and −77–82) at a distance of 23 nucleotides from each other.

In addition, one creA site (in the 5′–3′ direction, −400–405) was found in the promoter region and two differently directed XRE elements in the same place of the promoter region not far from gene start (−95–100 and −99–94).

MRE (metal responsive element) and ARE (antioxidant stress response element) sites were not found in the promoter region of the investigated gene. However, the one STRE element (“stress response element”) was identified in the 5′–3′ direction at the region of −167–171 bp. Also, two ACE sites regulated by protein factor activated in the presence of monovalent Cu+ and Ag+ ions were found in the 3′–5′-direction (−368–359 and −391–382 bp) at a distance of 14 bp from each other. In addition, two differently directed ATATC sequences were located at the distances of −379–383 and −427–423 bp from the laccase gene.

It should be noted that the promoter regions of laccase genes can include more than 1000 bp [45]. Thus, the promoter regions of the laccase gene of *C. geniculata* VKM F-3561 (461 bp) determined in this work is highly likely a partial sequence, which suggests an incomplete picture of all available regulatory elements of the gene.

The absence of a significant effect of Cu^2+^ ions on the induction of alkaliphilic laccases of the fungus *C. geniculata* VKM F-3561 is consistent with the absence of the corresponding MRE elements in the studied promoter region of the laccase gene. At the same time, the presence of two ACE elements in the promoter region suggests a possible regulation of transcription of the laccase gene by monovalent copper ions, which opens up the possibility for further optimization of laccase production during submerged cultivation of the fungus *C. geniculata* VKM F-3561.

It can also be assumed that alkaliphilic laccases of the fungus *C. geniculata* VKM F-3561 can be induced under conditions of nitrogen restriction, due to the presence of three NIT2-GATA sites, two of which are at a distance of 20 bp, and two NIT2-TATCDH sites at a distance of 20 bp from each other in the promoter region, which can be used in the future to optimize the yield of the target enzyme.

The presence of only two PacC regulatory sites does not give full confidence in the sensitivity of the promoter region of the *C. geniculata* VKM F-3561 laccase gene to high pH, since such sensitivity, as shown for laccase gene of *Fusarium oxysporum*, is achieved by the presence of at least three PacC elements in the promoter region [44].

As in the majority of fungal laccase genes [45], 14 repeated copies of the HSE sequences were found in the promoter region of the studied laccase gene of *C. geniculata* VKM F-3561, what indicates that temperature stress maybe involved in the regulation of these genes.

The presence of XRE elements in the promoter region of the studied gene in the absence of induction of the alkaliphilic laccase activity of the fungus by the tested aromatic inducers may suggest either an inappropriate regulation or regulation by other untested aromatic xenobiotics. Unfortunately, there is not enough information about this regulation, and more studies are required to understand the mechanism of transcription regulation of fungal laccase genes by different xenobiotics.

### 3.9. Homological Model of the Laccase of C. geniculata VKM F-3561 and Structural Analysis

The BLAST web service and the PDB database were applied to search for the *C. geniculata* VKM F-3561 laccase homologues with a known crystal structure, which were used as templates for calculating the homological model of the studied laccase structure using the Modeller 10.1 software package.

**Figure 7:**
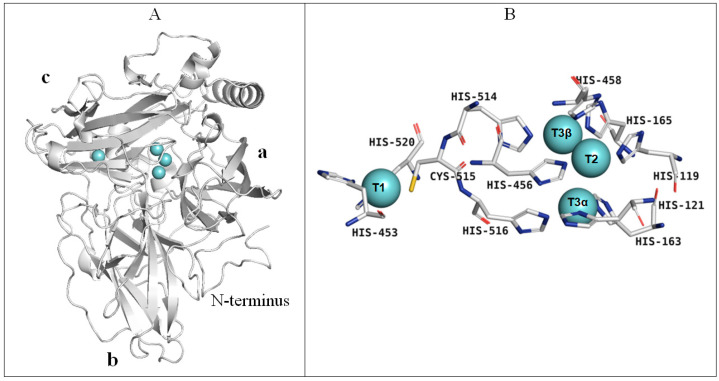
The structure of the laccase of *C. geniculata* VKM F-3561 calculated by homology modeling: (**A**)—the overall structure of the laccase molecule, (**B**)—the laccase copper sites. Copper atoms are marked in cyan.

According to the obtained model the laccase molecule of the *C. geniculata* VKM F-3561 expressed at the peak of the fungal alkaliphilic laccase activity had a typical fungal laccase structure, consisting of three cupredoxin-like domains (a, b, and c) and containing four copper ions in T1- and T2/T3-active sites located between domains a and c (Figure 7A,B).

**Figure 8:**
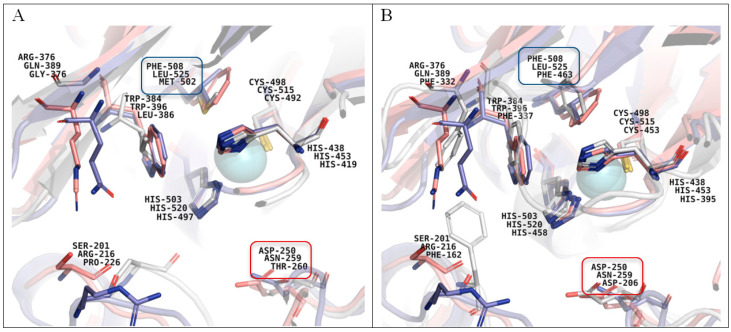
Superposition of the T1 centers of the laccases from *M. roridum* VKM F-3565 (top caption, salmon texture), *C. geniculata* VKM F-3561 (middle caption, violet purple texture), laccase-like bacterial protein CotA from *Bacillus subtilis* (RCSB PDB acc. no.: 1GSK, lower signature, gray structure, (**A**) or laccase from the fungus *Trametes versicolor* (RCSB PDB acc. no.: 1KYA, lower signature, gray structure, (**B**). The position of the axial methionine residue located near the T1-copper coordination sphere of bacterial laccases [84] is highlighted in blue. The position of the aspartate residue involved in the deprotonation of phenolic substrates in the T1-center cavity of the laccase of *Trametes versicolor* [86] is marked by the red frame.

In contrast to the alkaliphilic laccase of *M. roridum* VKM F-3565 [28], the subunit of *C. geniculata* VKM F-3561 laccase had a long N-terminus containing an increased amount of proline residues and protruding from the overall structure (Figure 7A and Figure 8) possibly involved in the formation of a multisubunit complex, which is consistent with the high molecular weight of *C. geniculata* VKM F-3561 laccases (1035 and 870 kDa) isolated at the peak of fungal alkaliphilic laccase activity.

The copper ion of the T1 site in the laccase homology model was coordinated by two histidine (His428, His 495) and one cysteine (Cys490) residues (Figure 7B). The cysteine residue transferring an electron from the T1-copper to the T3-copper atoms was further linked to two histidine residues, each of which coordinated T3α- (His491) or T3β-copper ions (His489). Each copper ion of the T3-cluster was coordinated, in turn, by three endogenous histidine residues. The T2-copper ion was adjacent to the T3-copper ions and was coordinated by two histidine residues (Figure 7B), as in all known laccases.

The superposition of the 3D structures of the investigated laccase, the alkaliphilic laccase of *M. roridum* VKM F-3565, the bacterial laccase-like protein CotA from *Bacillus subtilis*, and the laccase of the basidiomycete *Trametes versicolor*, is represented in Figure 8A. It is obvious that, in the position corresponding to the fourth axial T1-copper ligand (Met502) of the laccase-like protein CotA, the laccase of the *C. geniculata* VKM F-3561 contains a leucine residue (Leu525), while the *M. roridum* VKM F-3565 laccase has a phenylalanine residue (Phe508). These residues are typical for fungal laccases and presumably reflect the increased redox potential of their T1-copper [84,85].

The presence of a leucine residue in the axial position from the T1-copper atom in the structure of mutant forms of bacterial laccases led to temperature stability [87]. Possibly, the presence of Leu525 in the *C. geniculata* VKM F-3561 laccase is the reason of the increased stability of the alkaliphilic laccase-like oxidases isolated at the peak of alkaliphilic laccase activity with 100% retention of the laccase activity after 7 days of storage at room temperature.

In the structure of the *M. roridum* VKM F-3565 laccase, the aspartate residue was located at the same position as the Asp206 residue of the laccase of *Trametes versicolor* (Figure 8B) involved in the deprotonation of the substrate during its binding in the cavity of the T1-center [86]. At the same time, a polar asparagine residue was found in this position in the laccase molecule of *C. geniculata* VKM F-3561 as well as a polar threonine residue was observed in the bacterial laccase-like protein CotA from *Bacillus subtilis*. This probably impairs the substrate deprotonation and the accompanying electron transfer from the substrate molecule to the T1 copper ion.

**Figure 9:**
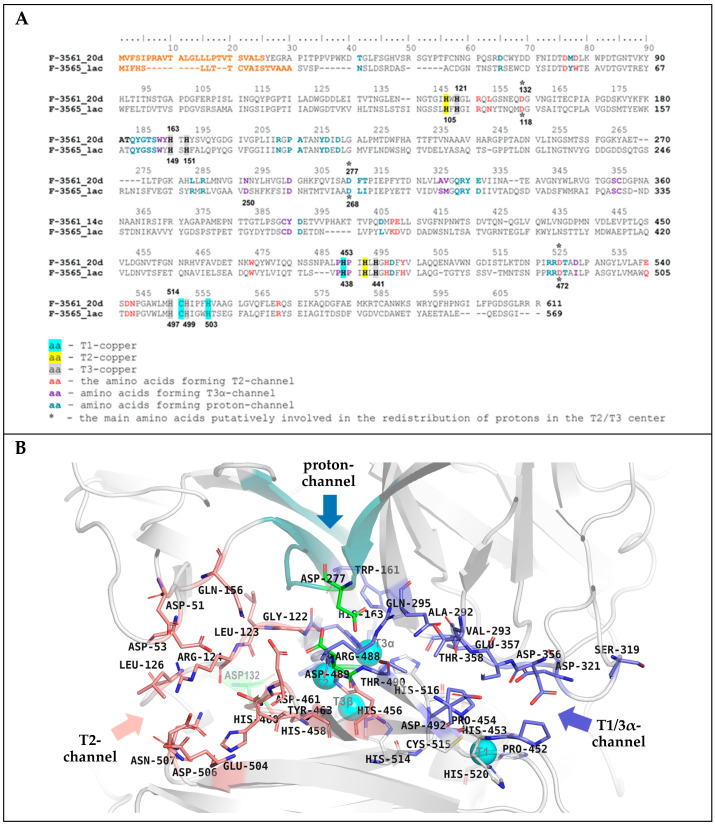
The alignment of amino acid sequences of the alkaliphilic laccases from *C. geniculata* VKM F-3561 (F-3561_20d) and *M. roridum* VKM F-3565 (F-3565_lac) containing signal peptides ((**A**), marked in orange) and T2-, T1/T3α-, and proton channels in the structure of *C. geniculata* VKM F-3561 laccase (**B**). The copper atoms are marked in blue. The aspartate residues presumably involved in proton transfer during a catalytic process are colored in light green.

As a result of the alignment of the primary amino acid sequences and the superposition of the three-dimensional structures of the laccases of *M. roridum* VKM F-3565 and *C. geniculata* VKM F-3561, two channels were found in the structure of the investigated laccase: the T1/T3α-channel was the entry of molecular oxygen to the T3- and T2-copper ions, and the T2-channel for the release of a water molecule, a product of the reduction of molecular oxygen during the catalytic reaction carried out by laccase (Figure 9). Both channels were combined in the T2/T3 center, which corresponds to the mechanism of the catalytic reaction of laccases [84].

The surface of the T2-channel of the laccase of *C. geniculata* VKM F-3561 was lined with amino acid residues Leu126, Asp132, Arg124, Asp53, Asp51, Gln156, Arg532, Asn507, Asp506, Glu504, Trp437, His460, Asp461, Asp489, Glu36 9, Tyr463 (Figure 9). Among these residues, Asp132, His460, Asp489 (the catalytic residue in all fungal laccases, corresponds to Asp472 in the laccase of *M. roridum* VKM F-3565), and Asn507 residues were conserved.

Comparison of residues lining the T2-channels of *C. geniculata* VKM F-3561 laccase and *M. roridum* VKM F-3565 laccase showed the same content of charged amino acids (Figure 9A). However, the aforementioned structure of the investigated laccase had more acidic and fewer basic residues, while *M. roridum* VKM F-3565 laccase contained more basic and less acidic residues. Thus, the T2-channel of the *C. geniculata* VKM F-3561 laccase will presumably be more negatively charged in neutral and alkaline medium with an insufficient number of protons.

The inner surface of the T1/T3α-channel of the *C. geniculata* VKM F-3561 laccase was formed by Pro452, Pro454, Asp492, Thr490, Arg488, Trp161, Gln295, Val293, Ala292, Thr358, Glu357, Asp356, Ser319, and Asp321. Residues Trp161, Gln295, Pro454, and Arg488 were conservative in the studied fungal laccases (Figure 9A) [28]. It should be noted that the higher content of charged acidic residues forming the T1/T3α-channel was observed in the laccase of *C. geniculata* VKM F-3561 than in the laccase of *M. roridum* VKM F-3565 (Figure 9B).

**Figure 10:**
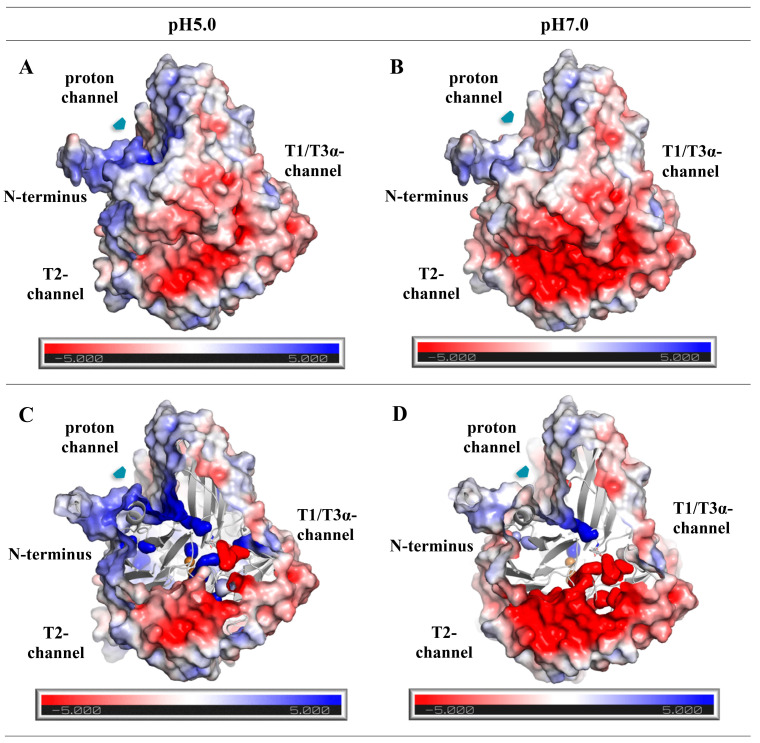
Connolly accessible surface representations colored according to electrostatic potential (−5 keV, red; +5 keV, blue) for laccase of *C. geniculata* VKM F-3561 calculated at pH 5.0 (**A**,**C**) and pH7.0 (**B**,**D**). (**A**,**B**) are external views, (**C**,**D**) are internal sections of the molecule. Copper atoms are highlighted in light brown.

The calculation of the electrostatic potential on the Connolly surface of the laccase of *C. geniculata* VKM F-3561 showed the presence of a proton channel near the catalytic residue Asp489 (Asp472 in laccase of the *M. roridum* VKM F-3565) positively charged both in an acidic and neutral medium, similar to the proton channels found in all alkaliphilic fungal laccases (Figure 10) [28].

The entrance of the proton channel was located near the N-terminus protruding from the molecule. The Asp277 residue of the *C. geniculata* VKM F-3561 laccase lining the bottom of the proton channel (similar to Asp268 in the alkaliphilic laccase of *M. roridum* VKM F-3565) was close to the Asp489 residue (Figure 10) [28].

Apparently, the similar structure of the proton channel and the amino acid composition of its bottom presumably act as a source of protons for compensation proton deficiency thereby supporting the function of the catalytic Asp489 and Asp132 residues of the laccase of *C. geniculata* VKM F-3561 in neutral and slightly alkaline conditions as in other alkaliphilic fungal laccases [28].

The catalytic triad of aspartate residues (Asp132/Asp118, Asp489/Asp472, and Asp277/Asp268 in the laccases of *C. geniculata* VKM F-3561 and *M. roridum* VKM F-3565, respectively) was found as a structural element presumably participating in the transfer of protons to the T2/T3-center during the catalytic reduction of molecular oxygen.

## 4. Conclusions

In the present work, the unique oligomeric alkaliphilic laccase-like oxidases with high biotechnological potential were isolated from the ascomycete *C. geniculata* VKM F-3561 and characterized for the first time. The enzymes were able to catalyze the oxidation of phenylpropanoids and phenolic compounds under neutral environmental conditions with the concomitant formation of potentially pharmacologically and industrially valuable di-, tri-, and tetrameric products. We demonstrated the presence of a proton channel in the *C. geniculata* VKM F-3561 laccase, which is similar to the proton channel of fungal alkaliphilic laccases of a known structure, providing the functional activity of the enzymes in a neutral-alkaline medium. The preparation and characterization of recombinant alkaliphilic laccase will be the subject of further research.

## Figures and Tables

**Table 1:** Physicochemical and kinetic properties of the purified oxidases of *C. geniculata* VKM F-3561 strain. At least three experiments were performed for calculating each kinetic parameter.

Substrates	Oxidase I				Oxidase II			
	pH- Optimum	*K*_m_, µM	*k*_cat_, min^−1^	*k*_cat_/*K*_m_, min^−1^ µM^−1^	pH- Optimum	*K*_m_, µM	*k*_cat_, min^−1^	*k*_cat_/*K*_m_, min^−1^ µM^−1^
ABTS	2.0	280.0	10758.0	38.4	2.0	152.0	8400.0	55.3
syringaldazine	7.5	2.3	12364.0	5375.7	7.0	46.0	41040.0	892.2
2,6-dimethoxyphenol	7.5–8.0	26.0	858.0	33.0	7.5–8.5	108.0	840.0	7.8
ferulic acid	7.0	37.6	48.6	1.3	6.8	51.0	885.0	17.4
coniferyl alcohol	7.0–8.0	130.0	1793.0	13.8	7.5	125.0	1472.0	11.8

All kinetic constants were obtained with error < 10%. The *K*_m_ values were measured using ABTS as a substrate in 20 mM Na-acetate buffer (pH 5.0), with the other substrates were measured in 50 mM Tris-HCl buffer (pH 7.2).

**Table 3:** List of the identified products of phenylpropanoids and phenolic compounds transformation by oxidases of the fungus *C. geniculata* VKM F-3561.

№	Coloring		TLC (11 cm), *R*_f_	Mass Spectrometric Analysis (Probable Structure), The Intensity of Fragment Ions is Indicated in Parentheses, %
	By Benzidine Reagent	In Visible Light		
**Ferulic acid**				
1	light brown	light orange	0.0	[M+H]^+^637(50%), 581(100%)—a condensation product of at least 3 molecules of ferulic acid, with successive elimination during ionization of two fragments with *m*/*z* = 56 (CH_2_=C^+^–CH=O) and formation of a fragment ion *m*/*z* 525. ([M+H]^+^581(90%), 525(100%)—daughter ion (resistant to ionization), also stable as *m*/*z* 525)
2	brown	light brown	0.0	[M+H]^+^736(100%), 574(42%), 556(90%), 538(23%)—condensation product of 4 molecules of ferulic acid, with successive elimination of *m*/*z* = 162 and *m*/*z* = 18 or simultaneously *m*/*z* = 180 (ferulic acid) during ionization
**Coniferyl alcohol**				
3	no	orange	0.0	[M+H]^+^341(100%), 323(42%), 311(17%), 208(76%)—a polymer
4	no	no	0.07	[M+H]^+^341(58%), 323(98%), 311(27%), 175(16%), 161(100%), 137(25%)—a polymer
**Caffeic acid**				
5	dark brown	rufous	0.0	[M+H]^+^141(100%), 123(80%), 95(85%), 77(20%)—hydroxybenzyl alcohol with *m*/*z* = 140—(HO)_2_C_6_H_3_CH_2_OH
6	dark brown	rufous	0.0	[M-H]^−^153(%), 137(7%), 120(10%), 108(40%), 88(100%)—hydroxytyrosol with *m*/*z* = 154 (HO)_2_C_6_H_3_CH_2_CH_2_OH
**Guaiacol**				
7	no	light cherry	0.29	[M-H]^−^455(90%), 325(100%), 294(40%)—presumably a heterotrimer with *m*/*z* = 456
8	no	light cherry	0.29	[M-H]^−^473(60%), 458(10%), 413(100%), 365(15%)—presumably heterotri(tetra)mer with *m*/*z* = 474
9	light orange	light yellow	0.41	[M+H]^+^369(45%), 337(100%), 305(40%), 245(20%)—a product of polymerization of three molecules of guaiacol with elimination of 4 protons, *m*/*z* = 368. Sequential elimination of two methoxyl groups is observed. The same metabolite is detected in negative ions [M-H]^−^367(90%), 352(100%)
10	light orange	light yellow	0.41	[M+H]^+^247(95%), 215(100%), 183(35%)—a product of polymerization of two molecules of guaiacol with elimination of 2 protons, *m*/*z* = 246. The spectrum shows the sequential elimination of two methoxyl groups. The same metabolite is detected in negative ions—[M-H]^−^245(72%), 230(100%)
11	light orange	red-brown	0.45	[M+H]^+^489(100%), 457(80%), 425(50%), 366(10%), 259(100%), 213(40%), 185(10%)—presumably the condensation product of 4 molecules of guaiacol with the elimination of 8 protons, *m*/*z* = 488
12	light orange	red-brown	0.45	[M+H]^+^261(95%), 229(100%)—presumably a condensation product of 2 molecules of guaiacol with monohydroxylation and concomitant elimination of 4 protons (*m*/*z* = 260)
13	light orange	red-brown	0.45	[M-H]^−^275(80%), 260(100%), 247(20%)—a product of condensation of 2 molecules of guaiacol with a dihydroxyl derivative and the formation of two bonds
14	light orange	light orange	0.51	[M-H]^−^271(100%), 253(45%), 243(20%), 192(30%)—presumably methylated guaiacol dimer with a double bond (CH_2_)_2_), *m*/*z* = 272
15	light orange	light orange	0.51	[M-H]^−^245(100%), 244(51%), 230(90%), 229(65%)—a product of guaiacol condensation with the formation of one bond (124 × 2 = 248-H2) = 246
**3-methoxycatechol**				
16	dark brown	red-brown	0.0	[M+H]^+^317(70%), 259(100%) or [M-H]^−^315(100%), 297(35%), 271(70%)—presumably a dimer with *m*/*z* = 316
17	dark brown	red-brown	0.0	[M-H]^−^289(100%), 245(90%), 226(15%)—presumably a dimer of the parent compound, *m*/*z* = 290
18	dark brown	red-brown	0.0	[M-H]^−^181(55%), 139(100%)—putative methylation product ((CH_2_)_3_) of parent compound, *m*/*z* = 182

## Data Availability

The nucleotide and amino acid sequences of the laccase gene reported in this paper were deposited in GenBank database with accession numbers OR250480.1.

## Supplementary Materials

The following supporting information can be downloaded at: https://www.mdpi.com/article/10.3390/microorganisms11112698/s1, Table S1. Primers used in the work; Table S2. The scheme of purification of the *C. geniculata* VKM F-3561 oxidases (the activity was measured in the reaction with ABTS at pH 5.0); Figure S1. Dependence of an activity of the purified laccase-like oxidase I of *C. geniculata* VKM F-3561 on temperature (in reaction with ABTS as a substrate) and pH (in the reaction with ABTS, syringaldazine, 2,6-dimethoxyphenol, ferulic acid, and coniferyl alcohol); Figure S2. Dependence of an activity of the purified laccase-like oxidase II of *C. geniculata* VKM F-3561 on temperature (in the reaction with ABTS as a substrate) and pH (in reaction with ABTS, syringaldazine, 2,6-dimethoxyphenol, ferulic acid, and coniferyl alcohol); Figure S3. Nucleotide sequence of the laccase gene and translated amino acid sequence of the laccase from the fungus *C. geniculata* VKM F-3561.
