# Supplementary material for: Unusual Oligomeric Laccase-like Oxidases from Ascomycete Curvularia geniculata VKM F-3561 Polymerizing Phenylpropanoids and Phenolic Compounds under Neutral Environmental Conditions
Source: Microorganisms. 2023 Nov 3;11(11):2698. doi: 10.3390/microorganisms11112698 (PMC10673308; doi:10.3390/microorganisms11112698)
Supplement: Supplementary file 1 [file microorganisms-11-02698-s001.zip › microorganisms-2627105-supplementary.pdf]

Supplementary material, Table 1. Primers used in the work

Primers	Sequence	Annealing temperature, ºC
Lac2FOR	GGIACIWIITGGTAYCAYWSICA	49
Lac3REV	CCRTGIWKRTGIAWIGGRTGIGG	49
F3561-20d-SON1-GSP1-F	GATGGCAATGTCACCTTC	63
F3561-20d-SON1-GSP2-F	CGTCACGTGTTTGCAGTC	55
F3561-20d-SON2-GSP1-R	TCTTGCATGAACGGCAG	63
F3561-20d-SON2-GSP2-R	GCATGAAACCAGTCGGTC	65
F3561-20d-SON3-GSP1-R	GGCCACTTGAGGTCCATATCGGTGT	60
F3561-20d-SON3-GSP2-R	GTCATCGTACCAGCAGTCTCTTGAT	63
F3561-20d-Lcc-F	ATGGTCTTTTCAATCCCGAGGGC	60
F3561-20d-Lcc-R	CTAGCGACGACGCAGACCAGA	60

Supplementary material, Table 2. The scheme of purification of the *C. geniculata* VKM F-3561 oxidases (the activity was measured in the reaction with ABTS at pH5.0)

Purification step	Total protein, mg	Total activity, U	Specific activity, U/mg	Purification, fold	Yield, %
Culture liquid	380.0	184.0	0.3	1.0	100.0
DE-52					
oxidase I	45.0	27.0	0.6	2.0	14.7
oxidase II	73.0	109.5	1.5	5.0	59.5
Q-Sepharose					
oxidase I	10.3	21.6	2.1	7.0	11.7
oxidase II	18.1	103.2	5.7	19.0	56.1
Resource Q					
oxidase I	-	-	-	-	-
oxidase II	10.7	95.2	8.9	29.7	51.7
Superdex 200					
oxidase I	1.3	20.0	15.4	51.3	10.9
oxidase II	3.7	84.7	22.9	76.3	46.0

Supplementary material, Figure 1. Dependence of an activity of the purified laccase-like oxidase I of *C. geniculata* VKM F-3561 on temperature (in reaction with ABTS as a substrate) and pH (in the reaction with ABTS, syringaldazine, 2,6-dimethoxyphenol, ferulic acid, and coniferyl alcohol). The error bars represent the standard deviation.

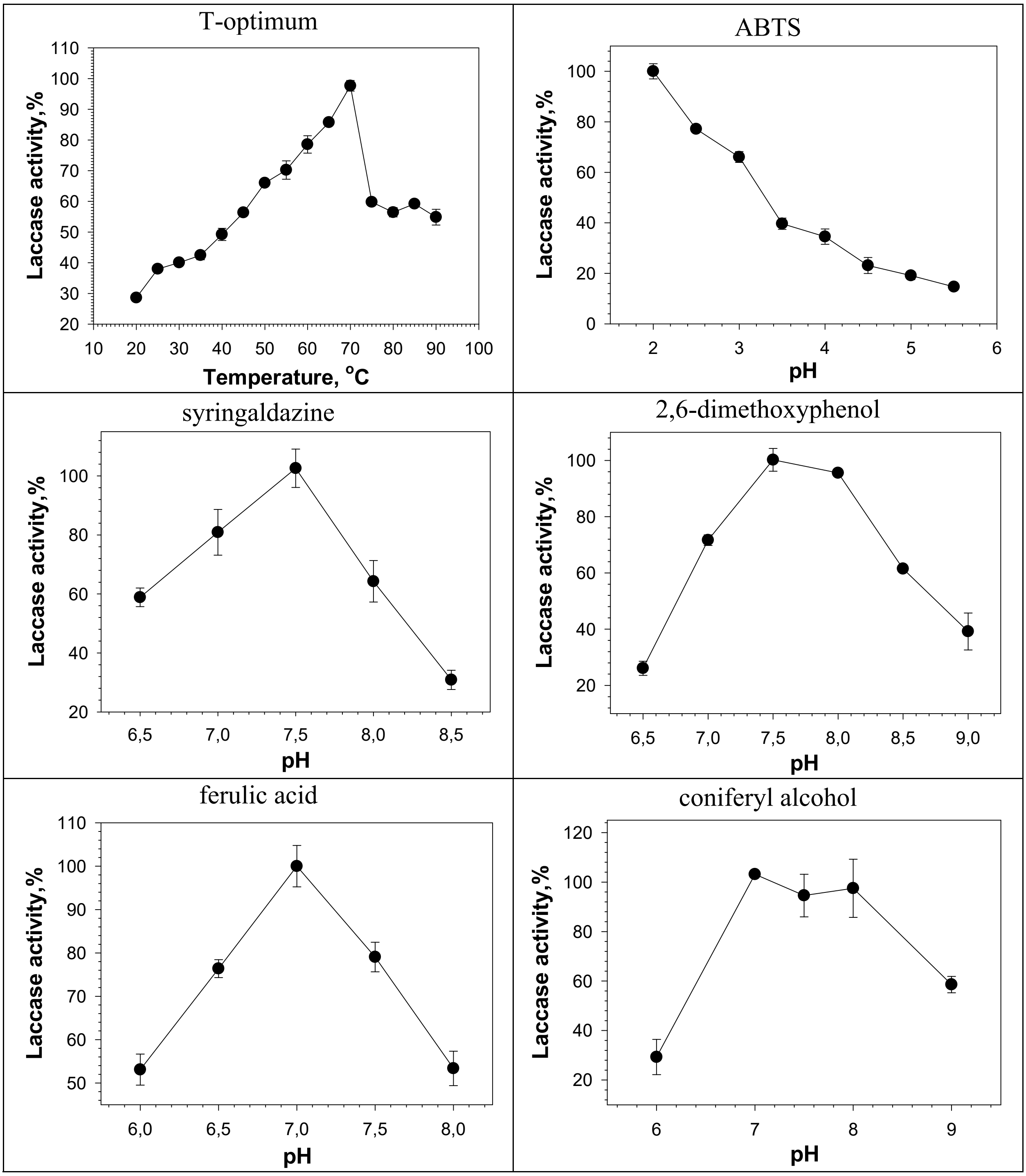

Supplementary material, Figure 2. Dependence of an activity of the purified laccase-like oxidase II of *C. geniculata* VKM F-3561 on temperature (in the reaction with ABTS as a substrate) and pH (in reaction with ABTS, syringaldazine, 2,6-dimethoxyphenol, ferulic acid, and coniferyl alcohol). The error bars represent the standard deviation.

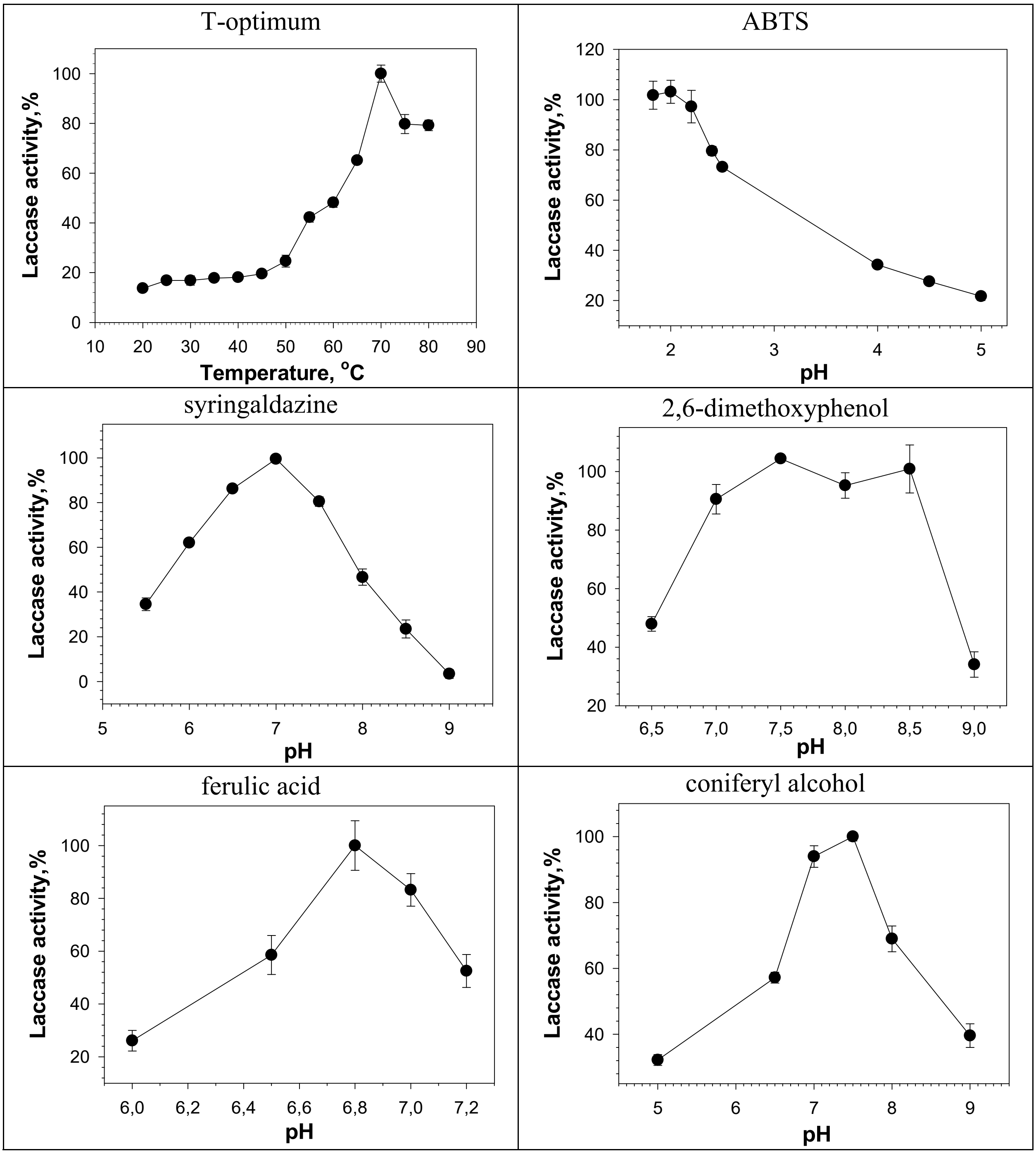

1 ATGGTCTTTTCAATCCCGAGGGCGGTCACCGCACTGGGGCTTCTCTTGCCTACTGTCACCTCTGTTGCCCTATCT 75

M V F S I P R A V T A L G L L L P T V T S V A L S

76 TATGAAGGCCGCGCACCCATAACACCTCCCGTCCCTTGGAAAGATACTGGACTCTTCTCTGGACACGTCAGCCGA 150

Y E G R A P I T P P V P W K D T G L F S G H V S R

151 TCAGGATATCCAACTTTTTGCAATAATGGTCCACAATCAAGAGACTGCTGGTACGATGACTTCAACATCGACACC 225

S G Y P T F C N N G P Q S R D C W Y D D F N I D T

226 GATATGGACCTCAAGTGGCCCGACACTGGCAATACAGTCAAGTACCACCTGACTATCACAAACTCTACCGGTGCT 300

D M D L K W P D T G N T V K Y H L T I T N S T G A

301 CCCGATGGTTTCGAGAGACCAATATCCTTGATCAACGGTCAATACCCAGGACCA**GTAAGTGTTATACAAACGATA** 375

P D G F E R P I S L I N G Q Y P G P

376 **CCGATAGTTCTTCTCTAATGAAATGTAG**ACAATACTGGCTGATTGGGGAGACGATTTGGAAATCACTGTTACCAA 450

интрон T I L A D W G D D L E I T V T N

451 CGGTTTGGAGAACAACGGAACAGGTATTCATTGGCATGGATTGCGACAACTCGGATCAAACGAGCAAGATGGAGT 525

G L E N N G T G I H W H G L R Q L G S N E Q D G V

526 GAACGGCATCACTGAATGCCCAATCGCGCCCGGCGACTCCAAAGTCTACAAGTTCAAGGCAACACAATACGGCAC 600

N G I T E C P I A P G D S K V Y K F K A T Q Y G T

601 CTCT**GTAAGTATCAGAAATTGTCTGACATGTTGAATGAAGACGACTGACCGTCACAG**TGGTATCACACGCACTAC 675

S интрон W Y H T H Y

676 TCCGTACAGTATGGCGACGGTATTGTAGGACCTTTAATCATCCGAGGACCTGCAACAGCAAACTACGATATTGAT 750

S V Q Y G D G I V G P L I I R G P A T A N Y D I D

751 CTCGGCGCCCTTCCAATGACCGACTGGTTTCATGCGACCACTTTCACTGTGAACGCTGCTGCCGTTCATGCAAGA 825

L G A L P M T D W F H A T T F T V N A A A V H A R

826 GGTCCTCCAACTGCGGACAACGTTCTGATCAACGGCTCCATGACTTCGTCTTTTGGTGGAAAATACGCTGAAACA 900

G P P T A D N V L I N G S M T S S F G G K Y A E T

901 ATTCTAACCCCTGGAAAGGCCCACTTGTTGCGCCTGATGAACGTTGGCATCAACAACTATCTCCATGTCGGTCTC 975

I L T P G K A H L L R L M N V G I N N Y L H V G L

976 GATGGACACAAGTTCCAGGTCATTTCCGCCGACTTTACCCCCATCGAACCATTCTACACCGACAACTTGGTTCTC 1050

D G H K F Q V I S A D F T P I E P F Y T D N L V L

1051 GCAGTCGGTCAACGCTATGAAGTCATCATTAATGCAACCGAGGCTGTTGGTAACTACTGGCTTCGCGTGGGTACT 1125

A V G Q R Y E V I I N A T E A V G N Y W L R V G T

1126 GGTGGTTCTTGTGATGGTCCCAACGCCAACGCGGCAAACATCAGGAGTATCTTCCGATACGCTGGCGCCCCTGCT 1200

G G S C D G P N A N A A N I R S I F R Y A G A P A

1201 ATGGAGCCCAACACGACTGGTACCCTTCCATCGGGCTGCTACGATGAGACTGTTGTGCCCCACGCCAAAACAACT 1275

M E P N T T G T L P S G C Y D E T V V P H A K T T

1276 GTTCCTCAGGACATGCCTGAATTGCTCAGTGTGGGCTTCAACCCCAACTGGACCAGCGACGTGACGCAAAACCAG 1350

V P Q D M P E L L S V G F N P N W T S D V T Q N Q

1351 GGCTTGGTTCAATGGCTTGTCAACGGCGACCCTATGAATGTCGACCTTGAAGTCCCCACACTACAGTCAGTATTG 1425

G L V Q W L V N G D P M N V D L E V P T L Q S V L

1426 GATGGCAATGTCACCTTCGGAAACAACCGTCACGTGTTTGCAGTCGACGAGACAAACAAG**GTATGTCATCTCTTG** 1500

D G N V T F G N N R H V F A V D E T N K

1501 **CGCTCGATATCATTCTTCCGTGACTAACACAACACTCCAG**TGGCAATACTGGGTCATCCAACAAAACAGCTCCAA 1575

интрон W Q Y W V I Q Q N S S N

1576 CCCAGCCCTTCCCCACCCCATCCACCTTCACGGCCACGACTTCTACGTCCTCGCCCAGCAAGAAAACGCAGTCTG 1650

P A L P H P I H L H G H D F Y V L A Q Q E N A V W

1651 GAACGGCGACATTTCCACCTTGAAGACCGACAACCCCATCCGTCGCGATACAGCCGACCTTCCCGCCAACGGCTA 1725

N G D I S T L K T D N P I R R D T A D L P A N G Y

1726 CCTGGTCCTTGCTTTCGAGTCTGACAACCCAGGTGCATGGCTCATGCACTGCCACATCCCTTTCCACGTTGCCGC 1800

L V L A F E S D N P G A W L M H C H I P F H V A A

1801 TGGTCTTGGCGTCCAGTTCCTGGAACGCCAGTCCGAAATCAAGGCCCAGGATGGATTTGCAGAGATGAAGAGGAC 1875

G L G V Q F L E R Q S E I K A Q D G F A E M K R T

1876 CTGTGCAAACTGGAAGTCTTGGCGCTACCAGTTCCATCCCAATGGTATCTTATTCCCTGGTGACTCTGGTCTGCG 1950

C A N W K S W R Y Q F H P N G I L F P G D S G L R

1951 TCGTCGCTAG 1960

R R *

Supplementary material, Figure 3. Nucleotide sequence of the laccase gene and translated amino acid sequence of the laccase from the fungus *C. geniculata* VKM F-3561. The intron is shown in bold. The putative signal peptide is shaded, possible N-glycosylation sites are in rectangles. The conservative amino acid sequences responsible for the binding of copper ions in the active site of the enzyme are underlined.
